# Experimental Study on Stress Monitoring of Sand-Filled Steel Tube during Impact Using Piezoceramic Smart Aggregates

**DOI:** 10.3390/s17081930

**Published:** 2017-08-22

**Authors:** Guofeng Du, Juan Zhang, Jicheng Zhang, Gangbing Song

**Affiliations:** 1 School of Urban Construction, Yangtze University, Jingzhou 434000, China; gfdu@yangtzeu.edu.cn (G.D.); 201671366@yangtzeu.edu.cn (J.Z.); 100995@yangtzeu.edu.cn; (J.C.Z.); 2 Department of Mechanical Engineering, University of Houston, Houston, TX 77204, USA

**Keywords:** Sand Filled Steel Tube Column (SFSTC), Smart Aggregate (SA), piezoceramics, calibration, impact loads, stress monitoring during impacts

## Abstract

The filling of thin-walled steel tubes with quartz sand can help to prevent the premature buckling of the steel tube at a low cost. During an impact, the internal stress of the quartz sand-filled steel tube column is subjected to not only axial force but also lateral confining force, resulting in complicated internal stress. A suitable sensor for monitoring the internal stress of such a structure under an impact is important for structural health monitoring. In this paper, piezoceramic Smart Aggregates (SAs) are embedded into a quartz Sand-Filled Steel Tube Column (SFSTC) to monitor the internal structural stress during impacts. The piezoceramic smart aggregates are first calibrated by an impact hammer. Tests are conducted to study the feasibility of monitoring the internal stress of a structure. The results reflect that the calibration value of the piezoceramic smart aggregate sensitivity test is in good agreement with the theoretical value, and the output voltage value of the piezoceramic smart aggregate has a good linear relationship with external forces. Impact tests are conducted on the sand-filled steel tube with embedded piezoceramic smart aggregates. By analyzing the output signal of the piezoceramic smart aggregates, the internal stress state of the structure can be obtained. Experimental results demonstrated that, under the action of impact loads, the piezoceramic smart aggregates monitor the compressive stress at different locations in the steel tube, which verifies the feasibility of using piezoceramic smart aggregate to monitor the internal stress of a structure.

## 1. Introduction

Piezoceramic material has characteristics of fast response, high sensitivity, good linearity, low cost, and sensing and actuating capacity. However, the material’s brittleness limits its application in concrete structures. For this reason, a piezoceramic smart aggregate was introduced, produced by embedding a waterproofed PZT (Lead Zirconate Titanate) patch with connecting wires into a concrete block [1]. At present, piezoceramic smart aggregates have been researched in the damage detection of civil engineering structures. Song et al. embedded the piezoceramic smart aggregate into a reinforced concrete beam to detect structural failure through destructive testing [1]. Feng et al. used SAs to monitor the damage of concrete piles [2]. Yang et al. used the structural mechanical impedance (SMI) extracted from the PZT electro-mechanical (PZT EM) admittance signature as the damage indicator [3]. A comparitive study on the sensitivity of the EM admittance and structural mechanical impedance to damage in concrete structures was conducted. Saravanan et al. compared the performance of various piezoceramic smart aggregates in the strength gain and damage states of concrete [4]. Yan et al. used distributive piezoceramic smart aggregates to monitor the health status of a concrete shear wall during a destructive test [5]. Gu et al. [6] embedded piezoceramic smart aggregates in a concrete block to study the change of the strength of the concrete block during the curing process. Nestorović et al. proposed a numerical modeling of the damage detection process in a concrete beam with piezoceramic smart aggregates [7]. Kong et al. used piezoceramic smart aggregates to monitor the freezing and thawing processes of soil [8] and the very early age of concrete curing [9]. Song et al. [10] proposed a new type of embedded piezoceramic sensor for the health monitoring of civil infrastructure. Jiang et al. presented a stress wave-based active sensing approach using piezoceramic transducers to monitor grouting compactness in real time [11]. Meng and Yan used the piezoceramic smart aggregate to identify the damage of a concrete beam, and proposed a damage index and studied damage probability based on wavelet packet analysis [12]. Song et al. summarized their pioneering research work in piezoceramic smart aggregates and innovative applications in concrete civil structures [13]. Yan et al. proposed a piezoceramic smart aggregate-based approach for the concrete compactness monitoring of concrete-filled steel tube (CFST) columns [14]. Liao et al. monitored damage on a concrete column using embedded piezoceramic smart aggregates under seismic loads [15]. Zhao et al. used the embedded piezoceramic smart aggregate in a concrete beam as an actuator, and surface-bonded piezoceramic patches as sensors to monitor the health of the concrete structure [16].

The internal stress state of the concrete structure plays an important role in the safety of concrete structures. At present, in a structural test, the structural stress is measured by attaching the strain gauge on the surface of the concrete structure. This monitoring method can only obtain the strain condition of the structural surface; however, it cannot effectively reflect the stress condition inside a structure [17,18]. In recent years, fiber optic sensors, piezoceramic smart aggregates and Polyvinylidene fluoride (PVDF) have been used as sensors to monitor structural stress conditions. Kerrouche et al. [19] used embedded Bragg grating optical fiber sensors to monitor the strain on carbon fiber polymer reinforcement (CFPR) rods. Ho et al. proposed a smart anchor plate, a simple but effective device that uses a fiber Bragg gratings (FBG) type optic sensor, to monitor the load level of the rock bolt [20]. Tennyson et al. installed long-gauge fiber optic sensors on bridges and pipelines to monitor their long-term structural integrity [21]. The results show that long-gauge sensors can provide an estimate of the maximum bending strain for beam-type structures. Huo et al. [22] used the Hopkinson rod to carry out the axial impact testing of concrete-filled steel tubular columns at high temperature. It was found that the temperature, impact velocity and steel content had a significant effect on the impact performance of CFST at high temperature. Meng et al. [23] embedded PVDF into concrete columns and impacted concrete columns with drop hammer. The reliability of PVDF as a stress sensor and the feasibility of implanting PVDF stress sensors into the structure were demonstrated. Liu et al. [24] encapsulated a piezoelectric patch with a concrete block and calibrated the sensor with an impact hammer. The results showed that the output voltage of the self-made piezoelectric sensor was proportional to the measured stress, and had good reproducibility. Narayanan et al. [25] used the electro-mechanical impedance measurements of bonded PZT patches to detect damage and substrate stress in a concrete structure. Hou et al. [26] conducted experiments to measure seismic compressive stress and shear stress in concrete structures using embedded compressive and shear smart aggregates. Xu and Wang [27] developed an embedded dynamic shear stress measurement sensor using a shear PZT. The sensitivity of SA was calibrated by using a free-falling weight. The results showed that the output voltage had good linear correlation with the external force, while the difference of sensitivity between different SAs was small. Chalioris et al. used embedded piezoceramic smart aggregate transducers and externally bonded piezoelectric patches to evaluate a shear-critical reinforced concrete beam [28].

In recent years, there have been some studies on sand columns [29,30,31]. However, there is no research on the internal stress monitoring of sand-filled steel tube column structures under impact loadings. In this paper, piezoceramic smart aggregates (SAs) were embedded into a quartz sand-filled steel tube column to monitor the internal structural stress during an impact. The filling of the thin-wall steel tube with quartz sand can prevent the premature buckling of the steel tube. The SAs were first calibrated using an impact force hammer. Tests were conducted to study the feasibility of monitoring the internal stress of a structure. These tests could lay the foundation for the stress monitoring of the piezoceramic smart aggregate buried in the concrete-filled steel tube column in the later stage.

## 2. Fabrication of Smart Aggregate

### 2.1. Selection of Piezoceramic Material and Fabrication of Smart Aggregate

**Figure 1:**
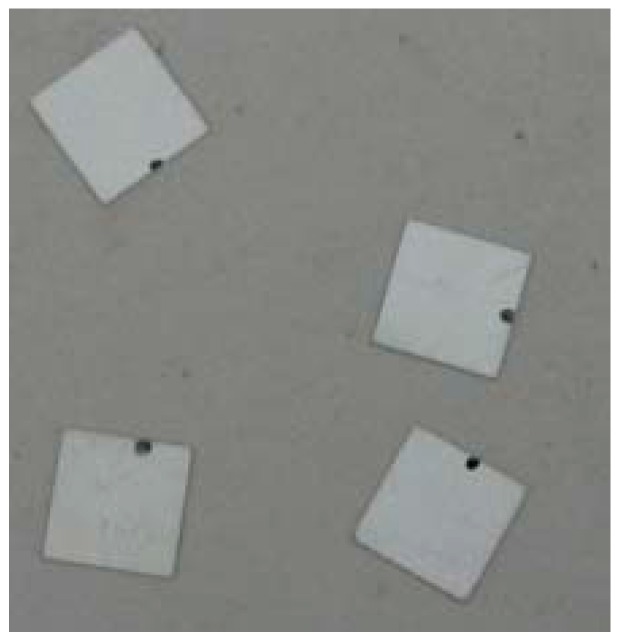
The piezoceramic patches.

Piezoceramic is the most critical component of the piezoceramic smart aggregate. Selecting the appropriate size and type of piezoceramics is an important prerequisite for the production of high-quality piezoceramic smart aggregates. At present, the most widely used piezoelectric material is Lead Zirconate Titanate (PZT). According to different physical and chemical properties, PZT can be divided into different types, including hard materials, such as PZT-2, PZT-4 (PZT-4A, PZT-4D, PZT-4E), PZT-8, and soft materials PZT-5 (PZT-5A, PZT-5D, PZT-5J, PZT-5H, PZT-5X). PZT-5H is highly sensitive to dynamic measurement and has good stability. It is widely used to build ultrasonic probes, electroacoustic transducers, sensors, accelerometers, manometers, buzzers, among others. In this paper, the PZT-5H type material is selected for this research due to its high sensitivity. The related parameters of PZT-5H are shown in Table 1. The size of the PZT patch is 10mm × 10mm × 0.3mm, as shown in Figure 1.

**Figure 2:**
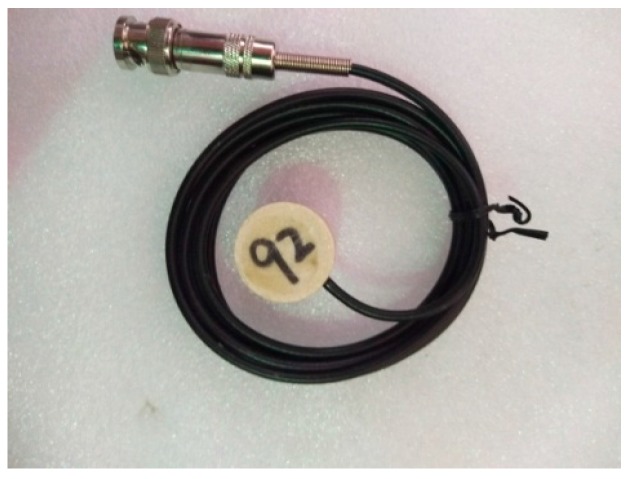
SA-1.

**Figure 3:**
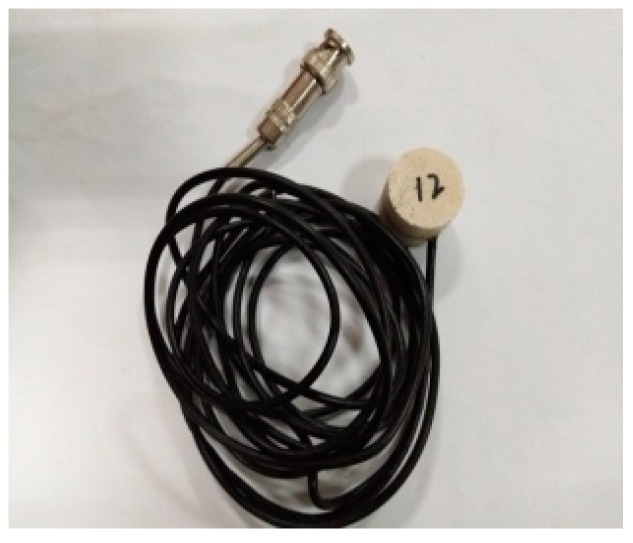
SA-2.

**Figure 4:**
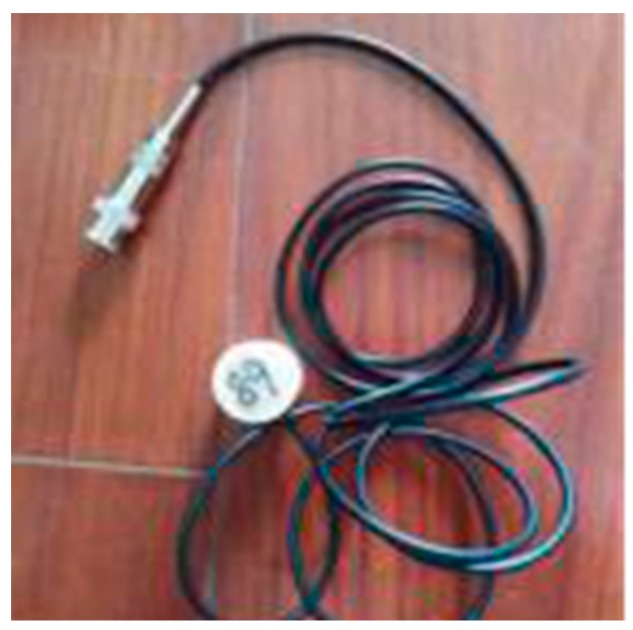
SA-3.

A piezoceramic smart aggregate is formed by sandwiching a PZT patch between two marble blocks. The piezoceramic smart aggregate has a cylindrical shape with a diameter of 25 mm and a height of 20 mm. A total of three piezoceramic smart aggregates were fabricated and are shown in Figure 2, Figure 3 and Figure 4.

### 2.2. Definition of Piezoceramic Smart Aggregate Sensitivity

The sensitivity of the piezoceramic smart aggregate is defined as the ratio of the output voltage to the applied stress, that is:

(1) Sensitivity (α)=output voltage(U)/stress(f)

For a compressive piezoceramic smart aggregate, as a result of piezoelectric effect, the piezoceramic will produce charge under the action of external force, and the relationship between the charge and the external force is

$$q=d_{33}F,$$

where $d_{33}$ represents piezoelectric constant of the piezoceramic and F represents the external force. According to this relationship, the following formula can be derived,

(2) $$U=\frac{d_{33}F}{C}$$

where U represents output voltage of piezoelectric material and C represents the feedback capacitance of the charge amplifier. An HK9209 charge amplifier is used in this research.

According to $F=f\cdot A_{\varepsilon }$, the following equation can be derived,

(3) $$\frac{d_{33}\cdot {\mathrm{fA}}_{\varepsilon }}{C}$$

where f represents uniform stress on piezoceramic patch and $A_{\varepsilon }$ represents the cross-sectional area of the piezoceramic patch.

From the sensitivity definition of the smart aggregate, the following equation can be derived,

(4) $$\alpha =\frac{d_{33}A_{\varepsilon }}{C}$$

Equation (4) indicates that the sensitivity of a piezoceramic smart aggregate is only related to the performance and size of the piezoelectric material and the switching capacitance of the charge amplifier. That is, each piezoceramic smart aggregate corresponds to a fixed sensitivity. The $d_{33}$ of the piezoceramic used in this experiment is 350 × 10^−12^ C·N^−1^, $A_{\varepsilon }$ is 100 mm^2^, and the capacitance of charge amplifier is 100 nF. Theoretical values of piezoceramic smart aggregate calculated by the (4) formula are shown in Table 2.

## 3. Calibration of Piezoceramic Smart Aggregate

**Figure 5:**
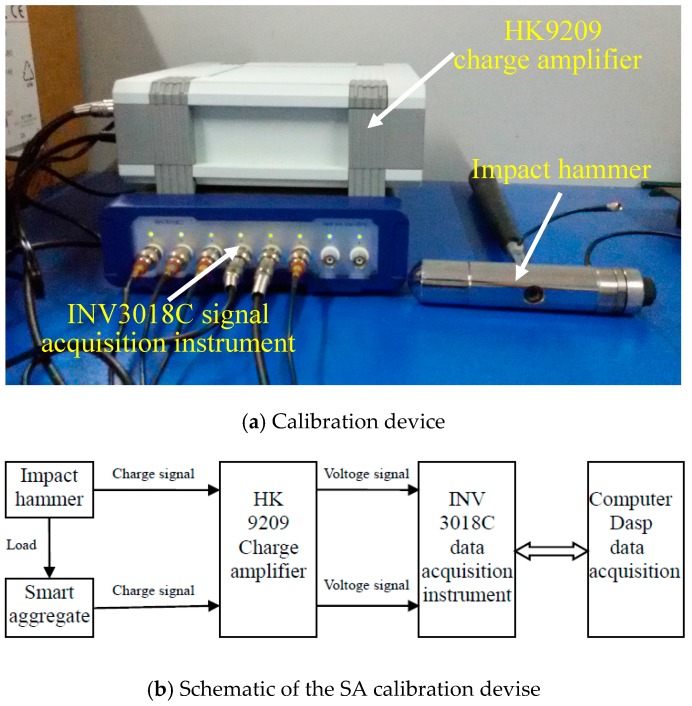
Smart aggregate calibration device under impact load of force hammer.

The calibration test equipment includes an impact hammer, a data acquisition system, and a charge amplifier, as shown in Figure 5. The charge amplifier converts a high-impedance charge signal produced by a piezoceramic sensor into a low-impedance voltage signal, and the output can be directly connected to a data acquisition system, a display instrument or a measuring device. Hence, it is necessary to use a charge amplifier. In the experiment, the gain of the HK9209 charge amplifier is adjusted to 100. The smart aggregate is fixed on a rigid plate, and the hammer is aimed at the center of the smart aggregate as much as possible, minimizing test error due to these causes.

**Figure 6:**
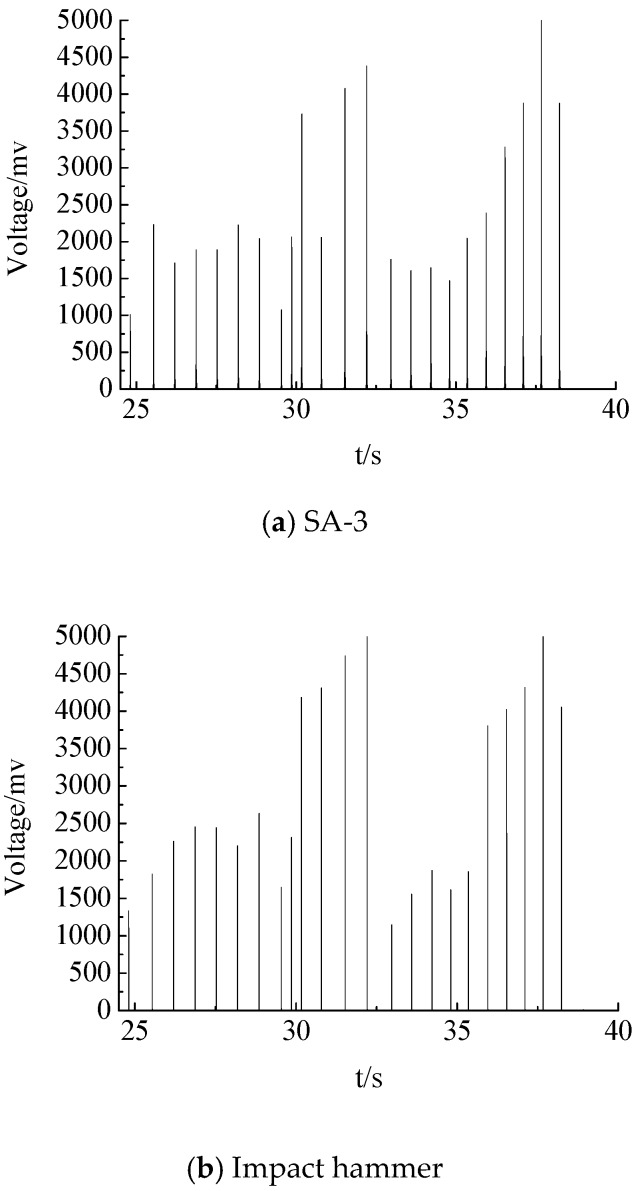

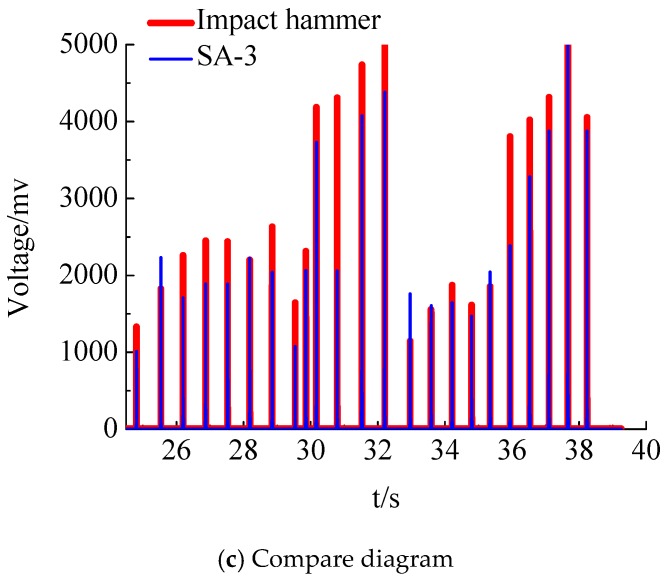
The waveform of the output voltage.

**Figure 7:**
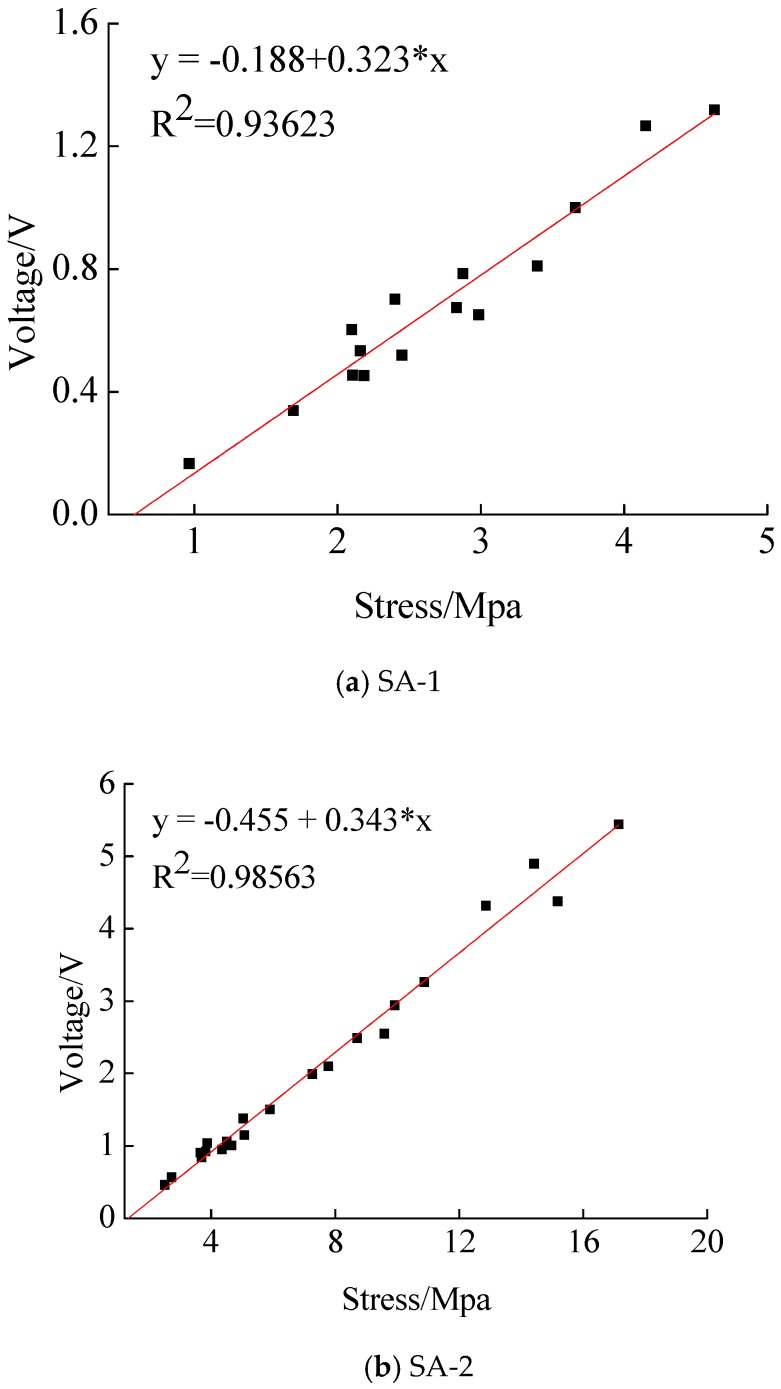

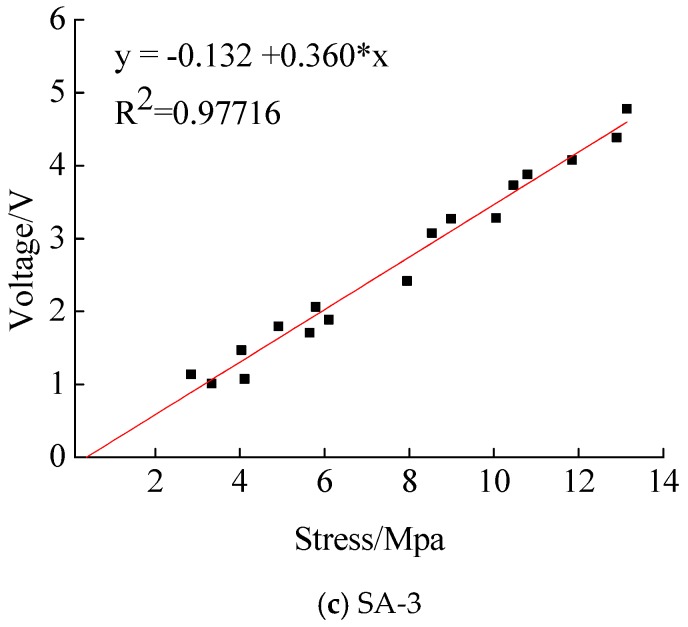
The calibration results of piezoceramic smart aggregates.

According to the waveforms of the output voltages of both the piezoceramic smart aggregate and the impact hammer, their waveforms in the time domain are comparable. Taking SA-3 as an example, as shown in Figure 6a,b, Figure 6a,b are placed in a diagram, as shown in Figure 6c. As can be seen from Figure 6c, the output voltage of the hammer varies with the force of the hammer; and the greater the hammering power, the greater the hammer output voltage. At the same time, the output voltage of the piezoceramic smart aggregate changes with the force of the hammer. Multiple load values and the corresponding peak values of output voltages are selected, and the calibration results of SA-3 under the impact load of the hammer can be fitted, as shown in Figure 7c. The slope of the curve represents the sensitivity of the compressive type piezoceramic smart aggregate. It can be seen that the sensitivity of SA-3 is = 0.360 V/MPa, and the squared correlation coefficient of the fitted line is above 97%. The calibration results of SA-1 and SA-2 using the same method are shown in Figure 7a,b, respectively. The sensitivity of each piezoceramic smart aggregate is shown in Table 2. Table 2 indicates that the sensitivity value of the piezoceramic smart aggregate is in good agreement with the theoretical value.

## 4. Stress Monitoring Test of Sand Filled Steel Tube Column Using Piezoceramic Smart Aggregate

### 4.1. Experiments

**Figure 8:**
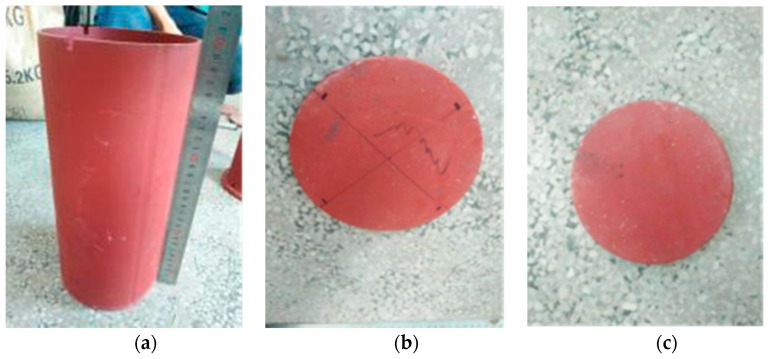
Test equipment: (**a**) Steel tube (wall thickness 300 mm); (**b**) Bottom plate (diameter 170 mm); (**c**) Cover plate (diameter 130 mm).

The outer diameter of the steel tube is 140 mm, its wall thickness is 3 mm, and its height is 300 mm. An upper cover plate with a diameter of 130 mm and a thickness of 10 mm was prepared, and the diameter of the cover plate was slightly smaller than the inner diameter of the steel tube, so that the quartz sand inside the tube can be constrained by the steel tube at the time of loading. A bottom plate with a diameter of 170 mm and a thickness of 10 mm was prepared. The test setup is shown in Figure 8. The mechanical properties of steel are shown in Table 3. The quartz sand with a diameter of 1–2 mm was poured into the steel tube and compacted through vibration. The hardness of quartz sand is higher, and it is used as the filler of the steel tube so that the strength of the smart aggregate placed in the interior is uniform, and the force condition is effectively reflected by the smart aggregate when the external load is applied.

**Figure 9:**
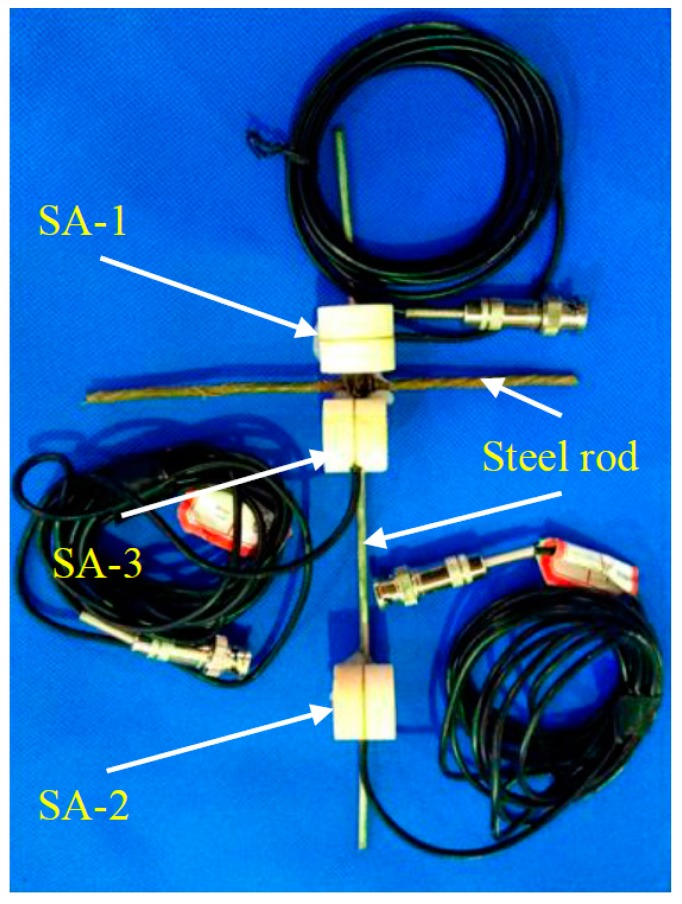
The smart aggregates prior to embedment.

**Figure 10:**
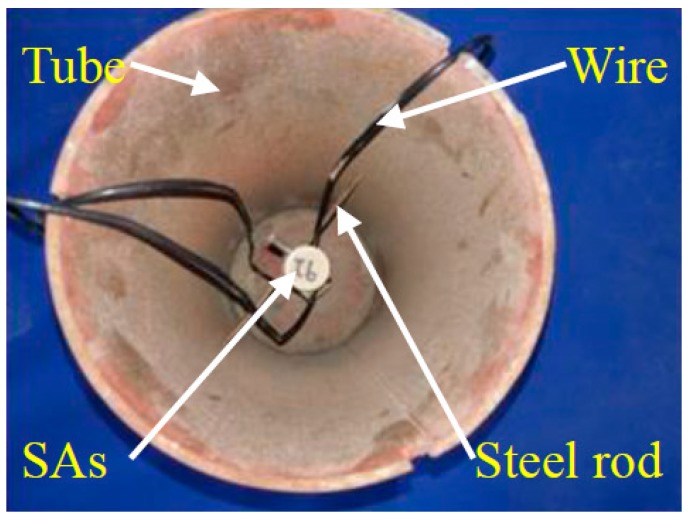
The placement of smart aggregates.

**Figure 11:**
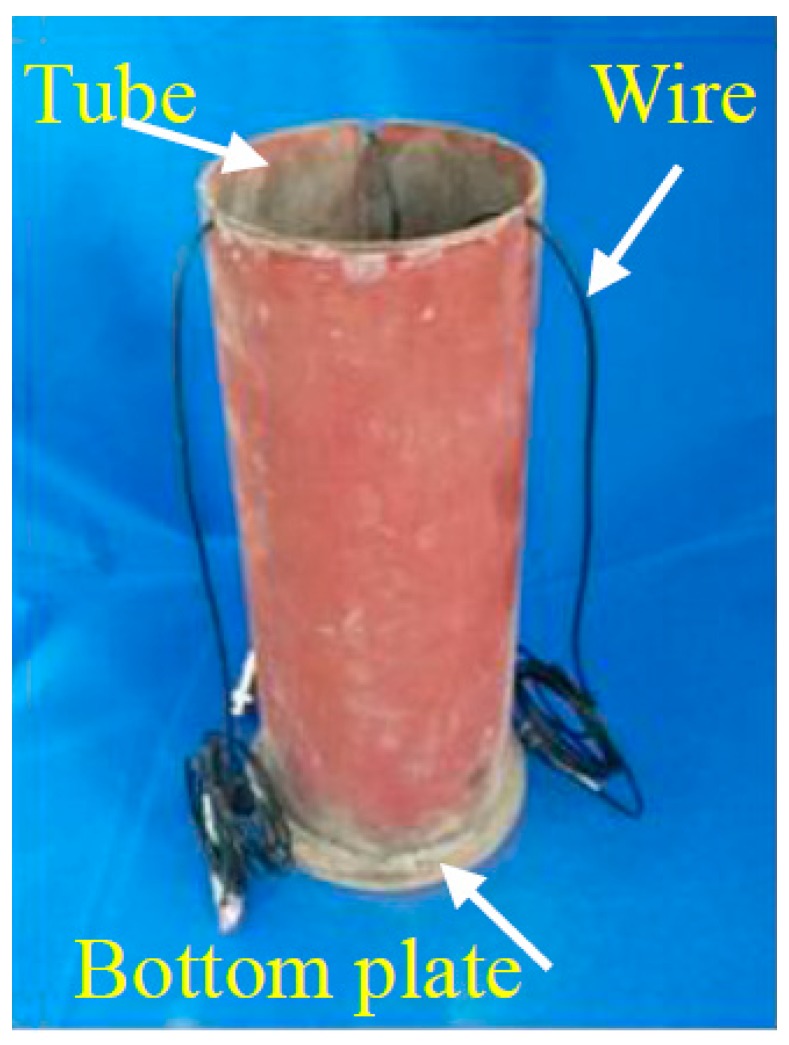
The steel tube.

**Figure 12:**
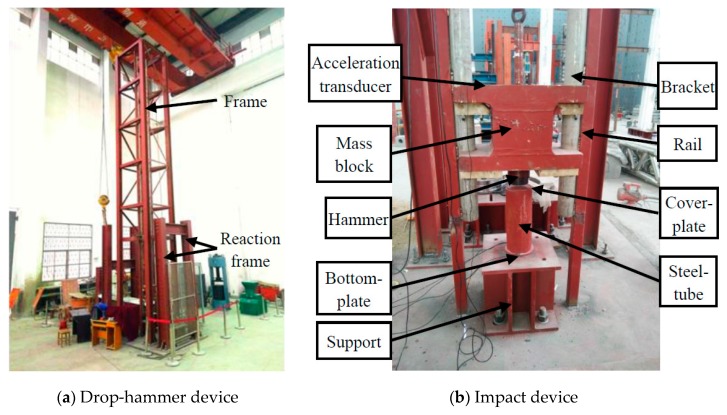

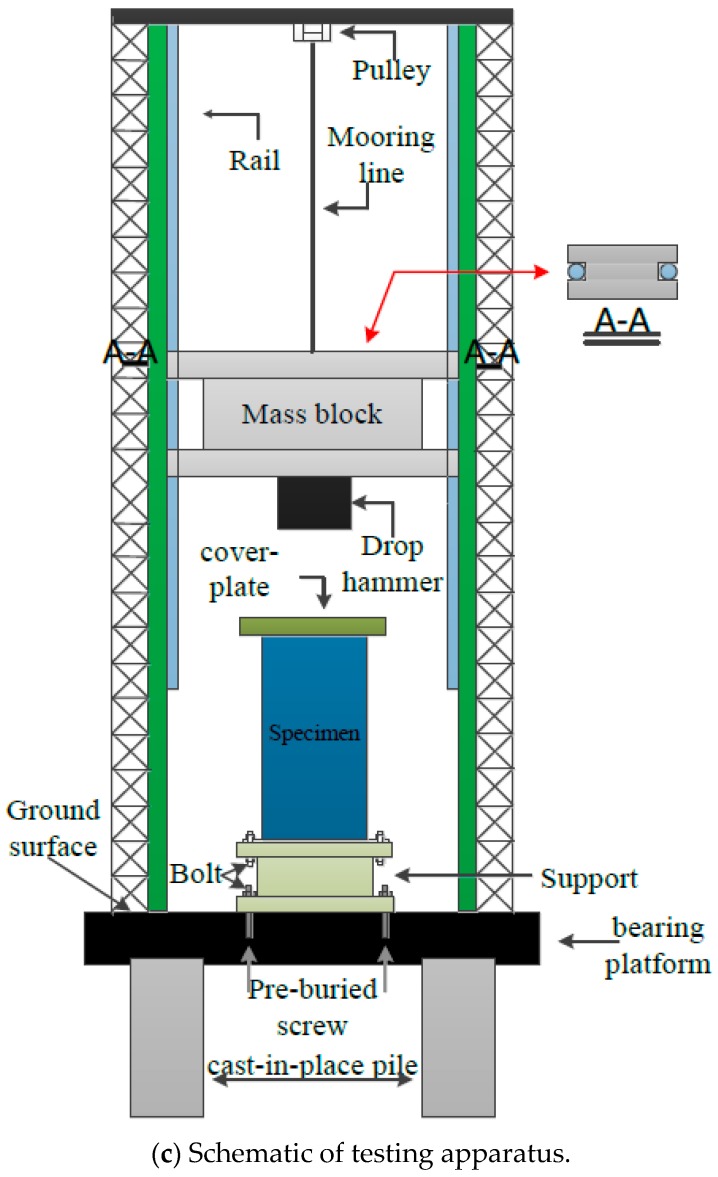
The impact test site.

Piezoceramic smart aggregates SA-1, SA-2, and SA-3, were attached to a steel rod with epoxy, as shown in Figure 9. SA-2 and SA-3 face laterally, and SA-1 faces vertically. The length of the steel rod is 180 mm. All the SAs were placed in the center of the steel tube vertically. The positions are shown in Figure 10 and Figure 11. Quartz sand was added to the inside of the tube and vibrated by the vibrating rod, and then the steel tube was covered by the cover plate. The stress levels of different locations were monitored by the SAs, embedded in the sand-filled steel tube column. SA-1 is used to monitor the axial stress. SA-2, SA-3 are used to monitor the lateral confining stress at different cross-sections. The sand-filled steel tube column was placed in the center of a drop hammer impact test system, as shown in Figure 12. The steel tube was impacted through the 339 kg drop hammer at heights of 0.25 m, 0.5 m, and 0.75 m, and the corresponding conditions were named case 1, case 2, case 3, respectively. An LC04 Accelerometer and LC0601 universal charge amplifier were mounted on the drop hammer to measure the impact force of the hammer. The SA output voltage was collected by the data acquisition system through the HK9209 charge amplifier, and the gain of the HK9209 charge amplifier was adjusted to 1.

### 4.2. Analysis of Test Results

#### 4.2.1. Time History Curve of Falling Hammer

**Figure 13:**
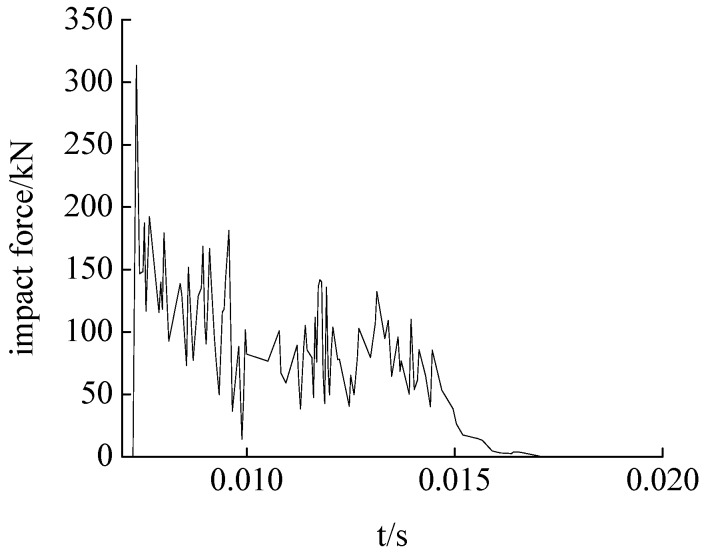
Time history curve of impact force under the second condition.

The test results show that the trend of the time history curve of the impact force of drop hammer in the three cases are comparable. The time history curve of the hammer impact force of case 2 is shown in Figure 13. It can be seen that the duration of the hammer impact is about 10 ms, during which the impact force rises rapidly to the maximum and then declines slowly to zero. The peak voltages of the three operating conditions are extracted and the hammer impact force are calculated, as shown in Table 4.

#### 4.2.2. The Responses of SAs

**Figure 14:**
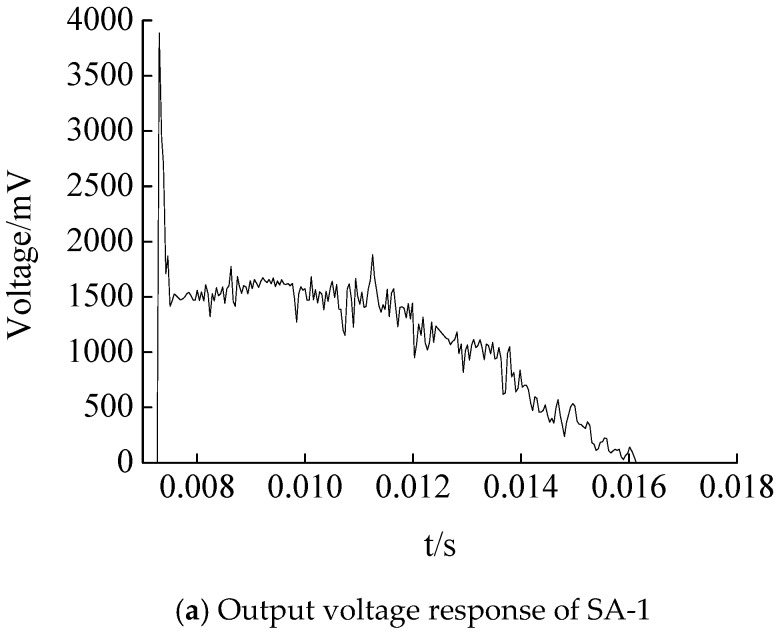

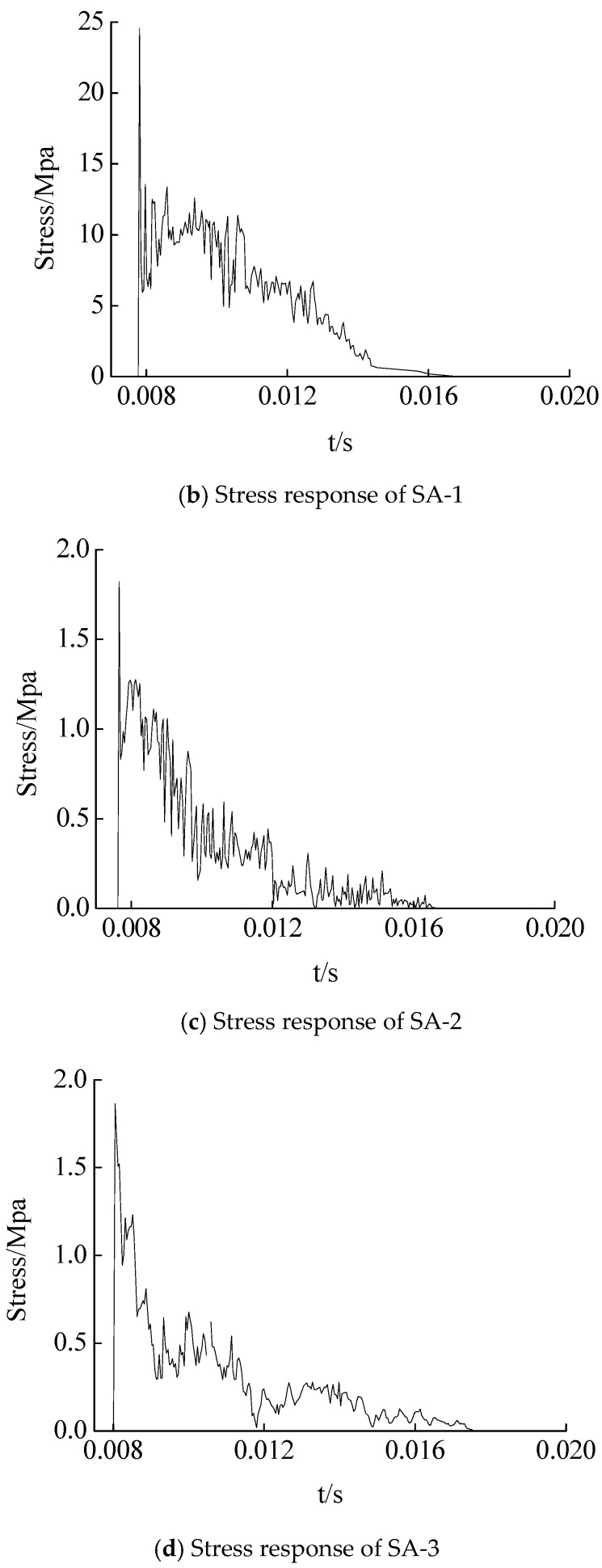
Smart aggregate responses.

The output voltage of SA-1 in case 2 is shown in Figure 14a, which shows that the response curve of the piezoceramic smart aggregate is basically synchronized with that of the falling hammer impact (Figure 13). The output voltage value rises rapidly to the peak value and then decreases to zero, and the response time is about 10 ms. The time history of the corresponding stress is shown in Figure 14b. Under the three conditions, the output voltage peak of a piezoceramic smart aggregate is extracted. According to the sensitivity value of the piezoceramic smart aggregate calibrated in the above test, the sensitivity value of the smart aggregate is 0.323 V/Mpa. Using Formula (1) to calculate the output stress peak value of the smart aggregate under the three conditions, the results were 17.13 MPa, 23.34 MPa, 25.198 Mpa, respectively. The test data are shown in Table 5. The output stress value of the smart aggregate increases with the increase of falling hammer impact energy. The test results support the feasibility of piezoceramic smart aggregate monitoring structural stress.

**Figure 15:**
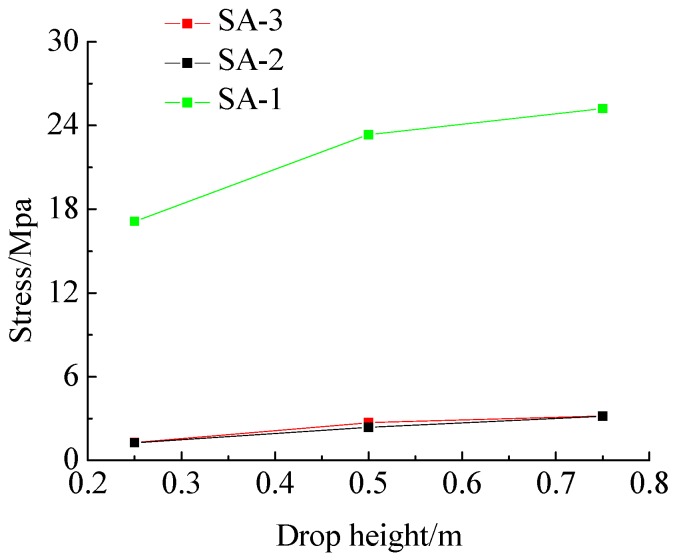
The relation between the Sas output stress and drop height.

Under case 2, the time history curves of the output voltage of SA-2 and SA-3 corresponding to stress are shown in Figure 14c,d, respectively. It can be seen that the stress time history is basically synchronized with that of the impact hammer. The maximum output voltage value and its corresponding stress values of piezoceramic smart aggregates obtained under different working conditions are shown in Table 5. It can be seen from Table 4 and Table 5 that the output stress values of piezoceramic smart aggregates increase with the increase of falling hammer impact energy under different working conditions. The results of internal lateral stress measurement by SA-2 and SA-3 show that the internal lateral stresses of the specimen increase with the increase of the impact energy. The confining stress measured by the SA-2 embedded in the bottom of the steel tube and SA-3 embedded in the middle of the steel tube under the same impact energy is not much different. The test results show that there is little difference between the internal stresses of different cross-sections of the structure. The authors have turned the results in Table 5 into a set of graphs, as shown in Figure 15, and the figure reveals that the greater the drop height, the greater the output value of piezoceramic smart aggregate, and there is a clear non-linearity in the relationship between sensor response and drop height. The reason for this phenomenon may be that the compressibility of sand can cause attenuation of impact energy after the impact of a sand filled steel tube column. 

## 5. Conclusions 

In this paper, embedded piezoceramic smart aggregates (SAs) were used to monitor the internal structural stress during impacts of a quartz sand-filled steel tube column. The filling of the thin-wall steel tube with quartz sand can prevent the premature buckling of the steel tube. Several conclusions can be drawn from this research: 

(1) Piezoceramic smart aggregate output voltage has good linear relationship with the external force in the sensitivity calibration test, and the sensitivity test value of piezoceramic smart aggregate is close to the theoretical value. This demonstrates that the sensitivity calibration test is reliable.

(2) Under the impact tests, the stress values of the piezoceramic smart aggregates embedded in the quartz sand-filled steel tube column increase with the increase in drop height. Under the same drop height, the confining stresses at different cross sections of the quartz sand-filled steel tube column are approximately the same. The feasibility of a piezoceramic smart aggregate to monitor the internal stress of a structure during an impact is verified.

## Figures and Tables

**Table 1:** Related performance parameters of PZT-5H.

Performance Category	Performance Value
Piezoelectric constant d_33_(10^−12^ C·N^−1^)	350
Electromechanical coupling factor (k_33_)	0.60
Density (kg/m^3^)	7600
Poisson ratio	0.35
Mechanical quality factor (Q_m_)	80
Relative permittivity ($\varepsilon_{r33}/\varepsilon_{0}$)	1600
Dielectric loss (tan δ)	0.025
Curie temperature (°C)	360

**Table 2:** Calibration result of smart aggregate under impact load.

Smart Aggregate	SA-1	SA-2	SA-3
Test sensitivity (v·MPa^−1^)	0.323	0.343	0.360
Theoretical sensitivity (v·MPa^−1^)	0.350	0.350	0.350
Squared correlation coefficient	92.9%	98.6%	97.7%

**Table 3:** The material properties of steel.

Steel Grade	Yield Strength *f_y_*/Mpa	Tensile Strength *f_y_*/Mpa	Density (kg/m^3^)	Poisson Ratio	Modulus of Elasticity E_S_/Mpa
Q235	289	396	7850	0.30	2.01 × 10^5^

**Table 4:** The test results of hammer’s impact force.

Drop Height (m)	Sensitivity (mv MPa^−1^)	Impact Speed (m/s)	Impact Energy (kJ)	The Output Voltage (v)	Maximum Impact Force (kN)
0.25	0.0199	2.21	0.83	0.77	267.35
0.50		3.13	1.66	1.07	370.08
0.75		3.83	2.49	1.16	400.93

**Table 5:** Test results of smart aggregate output.

Piezoceramic Smart Aggregate	Sensitivity (v MPa^−1^)	Drop Height (m)	Output Voltage (v)	Output Stress (MPa)
SA-1 (Upper axial stress)	0.323	0.25	0.39	17.13
		0.50	0.53	23.34
		0.75	0.57	25.20
SA-3 (Central ambient stress)	0.360	0.25	0.46	1.28
		0.50	0.98	2.72
		0.75	1.15	3.19
SA-2 (Bottom ambient stress)	0.343	0.25	0.46	1.27
		0.50	0.85	2.37
		0.75	1.14	3.17

## References

[1] Song G., Gu H., Mo Y., Mo Y.L., Hsu T.T.C., Dhonde H. (2007). Concrete structural health monitoring using embedded piezoceramic transducers. Smart Mater. Struct..

[2] Feng Q., Kong Q., Song G. (2016). Damage detection of concrete piles subject to typical damage types based on stress wave measurement using embedded smart aggregates transducers. Measurement.

[3] Yang Y., Hu Y., Lu Y. (2008). Sensitivity of PZT impedance sensors for damage detection of concrete structures. Sensors.

[4] Saravanan T.J., Balamonica K., Priya C.B., Reddy A.L., Gopalakrishnan N. (2015). Comparative performance of various smart aggregates during strength gain and damage states of concrete. Smart Mater. Struct..

[5] Yan S., Sun W., Song G., Gu H., Huo L., Liu B., Zhang Y. (2009). Health Monitoring of Reinforced Concrete Shear Walls Using Smart Aggregates. Smart Mater. Struct..

[6] Gu H., Song G., Dhonde H., Mo Y.L., Yan S. (2006). Concrete early-age strength monitoring using embedded piezoceramic transducers. Smart Mater. Struct..

[7] Nestorović T., Stojić D., Marković N. Active Structural Health Monitoring of Reinforced Concrete Structures using Piezoelectric Smart Aggregates. Proceedings of the 8th European Workshop On Structural Health Monitoring (EWSHM 2016).

[8] Kong Q., Wang R., Song G., Yang Z.J., Still B. (2014). Monitoring the soil freeze-thaw process using piezocermic-based smart aggregate. Cold Reg. Eng..

[9] Kong Q., Feng Q., Song G. (2015). Water presence concrete in a concrete crack using smart aggregate. Int. J. Smart Nano Mater..

[10] Song S., Hou Y., Guo M., Wang L., Tong X., Wu J. (2017). An investigation on the aggregate-shape embeded piezoelectric sensor for civil infrastructure health monitoring. Constr. Build. Meter..

[11] Jiang T., Kong Q., Wang W., Huo L., Song G. (2016). Monitoring of grouting compactness in a post-tensioning tendon duct using piezoceramic transducers. Sensors.

[12] Meng Y., Yan S. (2012). Statistical Algorithm for Damage Detection of Concrete Beams Based on Piezoelectric Smart Aggregate. Transactions.

[13] Song G., Gu H., Mo Y.L. (2008). Smart aggregates: multi-functional sensors for concrete structures a tutorial and a review. Smart Mater. Struct..

[14] Yan S., Fu J., Sun W., Qi B., Liu F. (2014). PZT-Based Detection of Compactness of Concrete in Concrete Filled Steel Tube Using Time Reversal Method. Math. Probl. Eng..

[15] Liao W.-I., Wang J.X., Song G., Gu H., Olmi C., Mo Y.L., Chang K.C., Loh C.H. (2011). Structural health monitoring of concrete columns subjected to seismic excitations using piezoceramic-based sensors. Smart Mater. Struct..

[16] Zhao J., Bao T., Chen S., Kundu T. (2016). Smart Aggregate-Piezoceramic Patch Combination for Health Monitoring of Concrete Structures. J. Sens..

[17] Wu C.L., Kuo W.W., Yang Y.S., Hwang S.J., Elwood K.J., Loh C.H. (2009). Collapse of a nonductile concrete frame: shaking table tests. Earthq. Eng. Struct. Dyn..

[18] Xiao H., Li H., Ou J. (2011). Strain sensing properties of cement-based sensors embedded at various stress zones in a bending concrete beam. Sens. Actuators A Phys..

[19] Kerrouche A., Boyle W.J.O., Sun T., Grattan K.T.V., Schmidt J.W., Taljsten B. (2009). Strain measurement using embedded fiber Bragg grating sensors inside an anchored carbon fiber polymer reinforcement prestressing rod for structural monitoring. IEEE Sens. J..

[20] Ho S., Li W., Wang B., Song G. (2017). A load measuring anchor plate for rock bolt using fiber optic sensor. Smart Mater. Struct..

[21] Tennyson R.C., Banthia N., Rivera E., Huffman S., Sturrock I. (2007). Monitoring structures using long gauge length fibre optic sensors. Can. J. Civ. Eng..

[22] Huo J., He Y., Chen B. (2014). Experimental study on impact behavior of concrete-filled steel tubes at elevated temperatures up to 800 °C. Smart Mater. Struct..

[23] Meng Y., Yi W. (2011). Application of a PVDF based stress gauge in determining dynamic stress-strain curves of concrete under impact testing. Smart Mater. Struct..

[24] Liu Y., Xu B., Li L., Li B. (2012). Development of a stress sensor based on the piezoelectric lead zirconate titanate for impact stress measurement. Proc. SPIE.

[25] Narayanan A., Subramaniam K.V.L. (2016). Sensing of damage and substrate stress in concrete using electro-mechanical impedance measurements of bonded PZT patches. Smart Mater. Struct..

[26] Hou S., Zhang H., Ou J. (2012). A PZT-based smart aggregate for compressive seismic stress monitoring. Smart Mater. Struct..

[27] Xu B., Wang D. (2015). Development of Embedded PZT-Based Dynamic Shear Stress Sensors for Concrete Structures. Piezoelectr. Acoust..

[28] Chalioris C.E., Papadopoulos N.A., Angeli G.M., Karayannis C.G., Liolios A.A., Providakis C.P. (2015). Damage evaluation in shear-critical reinforced concrete beam using piezoelectric transducers as smart aggregates. Open Eng..

[29] Tandel Y.K., Solanki C.H., Desai A.K. (2014). Field behaviour geotextile reinforced sand column. Geomech. Eng..

[30] Peters D.J., Broos E.J., Gresnigt A.M., Es S.H.J.V. Local buckling resistance of sand-filled spirally welded tubes. Proceedings of the Twenty-Fifth International Society of Offshore and Polar Engineers.

[31] Maakaroun T., Najjar S.S., Sadek S. Effect of Sand Columns on the Load Response of Soft Clays. Proceedings of the International Foundation Congress and Equipment Expo.

